# MiR‐30‐5p suppresses cell chemoresistance and stemness in colorectal cancer through USP22/Wnt/β‐catenin signaling axis

**DOI:** 10.1111/jcmm.13968

**Published:** 2018-10-19

**Authors:** Shixiong Jiang, Dazhuang Miao, Muhong Wang, Jiachen Lv, Yihui Wang, Jinxue Tong

**Affiliations:** ^1^ Department of Colorectal Surgery Harbin Medical University Cancer Hospital Harbin China

**Keywords:** chemoresistance, colorectal cancer, MiR‐30‐5p, stemness, USP22, Wnt/β‐catenin signaling

## Abstract

Colorectal cancer (CRC) remains both common and fatal, and its successful treatment is greatly limited by the development of stem cell‐like characteristics (stemness) and chemoresistance. MiR‐30‐5p has been shown to function as a tumor suppressor by targeting the Wnt/β‐catenin signaling pathway, but its activity in CRC has never been assessed. We hypothesized that miR‐30‐5p exerts anti‐oncogenic effects in CRC by regulating the USP22/Wnt/β‐catenin signaling axis. In the present study, we demonstrate that tissues from CRC patients and human CRC cell lines show significantly decreased miR‐30‐5p family expression. After identifying the 3’UTR of USP22 as a potential binding site of miR‐30‐5p, we constructed a luciferase reporter containing the potential miR‐30‐5p binding site and measured the effects on USP22 expression. Western blot assays showed that miR‐30‐5p decreased USP22 protein expression in HEK293 and Caco2 CRC cells. To evaluate the effects of miR‐30‐5p on CRC cell stemness, we isolated CD133 + CRC cells (Caco2 and HCT15). We then determined that, while miR‐30‐5p is normally decreased in CD133 + CRC cells, miR‐30‐5p overexpression significantly reduces expression of stem cell markers CD133 and Sox2, sphere formation, and cell proliferation. Similarly, we found that miR‐30‐5p expression is normally reduced in 5‐fluorouracil (5‐FU) resistant CRC cells, whereas miR‐30‐5p overexpression in 5‐FU resistant cells reduces sphere formation and cell viability. Inhibition of miR‐30‐5p reversed the process. Finally, we determined that miR‐30‐5p attenuates the expression of Wnt/β‐catenin signaling target genes (Axin2 and MYC), Wnt luciferase activity, and β‐catenin protein levels in CRC stem cells.

## 1 INTRODUCTION

Colorectal cancer (CRC) is the third most commonly diagnosed cancer in the United States for men and woman, and it remains the second leading cause of cancer‐related death.^1^ Even though the incidence and mortality from CRC have declined substantially in the past few decades, these decreases are largely due to improvements in screening and diagnosis, rather than to treatment advancements.^2^ Nevertheless, response to first‐line therapy is a significant prognostic indicator, regardless of second‐line therapy success.^3^ It is therefore crucial to ensure that patients respond maximally to first‐line therapies and that therapeutic resistance does not develop. The fluoropyrimidine fluorouracil (5‐FU) is a first‐line adjuvant chemotherapeutic often given as part of a regimen with other cytotoxic drugs including irinotecan,^4^ oxaliplatin^5^ and leucovorin.^6^ Understanding the mechanisms of chemotherapeutic resistance to particular agents is crucial to developing treatment strategies that improve responses to first‐line regimens.^7^ In the present study, we explored the role of microRNA 30‐5p (miR‐30‐5p) in CRC progression and determined that it inhibits CRC stemness and chemoresistance by negatively regulating the ubiquitin‐specific peptidase 22 (USP22)/Wnt/β‐catenin signaling axis.

MiRNAs are short, non‐coding, evolutionarily conserved RNAs that post‐transcriptionally regulate gene expression, mainly by binding the 3’ untranslated regions (3'UTRs) of mRNAs.^8^ They were originally recognized for their indispensable roles in the maintenance of crucial cellular process, including cell fate determination, cell growthand stress responses.^9^ Increasingly, however, miRNAs are being appreciated for their effects on pathological processes, including oncogenesis and drug resistance.^10^, ^11^, ^12^ In fact, miRNA activity can function as a prognostic biomarker that predicts the response of CRC to neoadjuvant chemoradiotherapy.^13^ MiR‐30 is the microRNA precursor of the mature strands miR‐30‐5p and miR‐30‐3p. MiR‐30 as well as members of the miR‐30‐5p family (miR‐30a‐5p, miR‐30b‐5p, miR‐30c‐5p, miR‐30d‐5pand miR‐30e‐5p) have been shown to play crucial roles in breast cancer and have the potential to serve as cancer‐related biomarkers.^14^, ^15^ However, based on miRNA network studies, it is likely that miR‐30 and its mature strands act either anti‐ or pro‐oncogenically in other cancers as well.^16^ So far, the majority of research into the miR‐30 family has focused on miR‐30a, which has been shown to negatively regulate TGF‐β1‐induced epithelial‐to‐mesenchymal transition (EMT),^17^ increase cisplatin sensitivity of gastric cancer cells,^18^ and suppress oncogenesis and metastasis in CRC.^19^, ^20^ Interestingly, miR‐30a has also been shown to serve as both oncogene or onco‐suppressor in different cancer types.^21^ Apart from miR‐30a, miR‐30b‐5p functions as a tumor suppressor in renal cell carcinoma by inhibiting EMT, cell proliferationand metastasis.^22^ Given the potentially conflicting effects of the miR‐30 family on oncogenesis and the fact that the miR‐30‐5p mature strand has not been extensively explored in CRC, we decided to investigate the effects of miR‐30‐5p family members on CRC stemness and chemoresistance.

In our previous study,^23^ we found that ubiquitin‐specific peptidase 22 (USP22) promotes CRC stemness and chemoresistance through the Wnt/β‐catenin signaling pathway. In the present study, after determining that miR‐30‐5p expression is reduced in CRC tissues and cell lines, we identified the 3'UTR of USP22 as a binding site of miR‐30‐5p. USP22 positively regulates a number of oncogenic signaling pathways that cause a variety of lethal cancer phenotypes.^24^ In 2017, Li et al. demonstrated that USP22 also promotes EMT, thereby increasing CRC invasion and metastasis.^25^ Mechanistically, there is evidence that USP22 promotes cell cycle progression by increasing β‐catenin nuclear localization, which is necessary for Wnt pathway activation.^26^ The Wnt/β‐catenin pathway is an evolutionarily conserved signal transducer responsible for regulating a host of normal physiological processes such as cell proliferation, cell differentiationand cell polarity.^27^, ^28^ However, like many other signaling pathways, it also contributes to disease, including numerous cancers.^29^ Specifically in CRC, the Wnt/β‐catenin pathway promotes cancer stem cell (CSC) maintenance, tumorigenesisand chemoresistance.^30^, ^31^, ^32^ In the present study, we have determined that miR‐30‐5p not only targets USP22 but also attenuates the Wnt/β‐catenin pathway, thereby negatively regulating CRC stemness and chemoresistance.

## 2 MATERIALS AND METHODS

### 2.1 Tissue specimens

This study was approved by the Research Ethics Committee of the Harbin Medical University Cancer Hospital (Harbin, China). All patients provided written informed consent. Paired fresh primary CRC tissues and normal adjacent tissues were obtained from 30 patients who underwent surgery at the Affiliated Cancer Hospital of Harbin Medical University. The specimens were snapped into liquid nitrogen and stored at −80°C until processing.

### 2.2 Cell culture

Human CRC cell lines (Caco2, HT29, HCT15, HCT116, SW620 and SW480) and HEK293T were obtained from the Shanghai Institutes for Biological Sciences of the Chinese Academy of Sciences or ATCC. Cells were cultured at 37°C in 5% CO_2_ atmosphere in RPMI‐1640 medium (Hyclone, Logan, UT, USA), supplemented with 10% bovine calf serum (Hyclone) and 2 mmol/L^−1^ L‐glutamine. Caco2 and HCT15 CD133 + cells were cultured in RPMI‐1640 medium, supplemented with B27, heparin, N2 supplement, 20 ng/mL epidermal growth factor (EGF) and 20 ng/mL basic fibroblast growth factor (bFGF). Lipofectamine 3000 was used for plasmid and miRNA transfection according to the manufacturer's instructions.

### 2.3 Reverse transcription PCR (RT‐PCR)

Total RNA was isolated from CRC tissues and cell lines using Trizol reagent (Invitrogen, Carlsbad, CA, USA) and reverse transcribed into cDNA using Superscript First‐Strand Synthesis System (Invitrogen) according to the manufacturer's instructions. Expression of miRNA was detected using the TaqMan^®^ microRNA Reverse Transcription Kit (Applied Biosystem, Foster City, CA, USA), in accordance with the manufacturer's protocol. *GAPDH* or snRNAU6 were used as an internal control. The primers were used as previously described.^23^ PCR conditions were: initial denaturation at 95°C for 2 minutes, followed by 30 cycles of amplification at 95°C for 30 seconds, 55°C for 45 seconds and 72°C for 1 minute, and a final extension at 72°C for 15 minutes. The fold‐change was calculated using 2^−ΔΔCt^ method.

### 2.4 Luciferase assay

Wild‐type and mutant USP22 3'UTR was constructed into the psiCHECK2 reporter vector. HEK 293T cells were seeded and cultured in 96‐well plate overnight. Then the cells were transfected with wild‐type USP22 reporter plasmid and miR‐30‐5p or miR‐control. After 48 hours of transfection, luciferase activity was measured using the Dual‐Luciferase Reporter Assay System (Promega, Madison, WI, USA). Relative luciferase activity was expressed as the ratio of firefly luciferase activity to *Renilla* luciferase activity.

### 2.5 Western blot

Total protein from CRC tissues and cells was extracted in a lysis buffer consisting of 20 mM Tris–HCl (pH 7.5), 2 mmol/L^−1^ EDTA, 150 mM NaCl, 1% Triton X‐100, and protease inhibitors. Protein was analysed in the supernatant by the Bradford method (BioRad, Hercules). Proteins in all samples were separated by SDS‐PAGE (10%) and transferred onto nitrocellulose membrane. Membranes were probed with primary antibodies overnight at 4°C. After washing, the membranes were incubated with the HRP‐conjugated secondary antibody for 1 hour. The following antibodies were used: antibodies against USP22 and β‐catenin were from Abcam (Cambridge, MA, USA); antibodies against MYC, Sox2 and Axin2 were from Cell Signaling Technology (Danvers, MA, USA); and antibodies against CD133 were from Santa Cruz Biotechnology (Santa Cruz, CA, USA).

### 2.6 CD133 + cell isolation

CD133 + CRC cells were isolated from the Caco2 and HCT15 cell lines using magnetic‐activated cell sorting (MACS; Miltenyi, Bergisch Gladbach, Germany), according to the manufacturer's instructions. Briefly, CRC cells were collected and centrifuged for 5 minutes. The supernatant was removed and 20 μL CD133 microbeads were mixed in and incubated for 15 minutes at 4°C. The cells were washed twice to remove the uncombined microbeads. The CD133 + cells were isolated by a magnetic separation column. In order to verify the efficiency of cell isolation, the isolated cells were stained with CD133‐PE and analysed by flow cytometry (BD Biosciences, San Jose, CA, USA).

### 2.7 Sphere formation assays

CRC cells (1 × 10^3^ cells/well) were plated in 6‐well plates with ultra‐low adherence (Corning, Corning, NY, USA) and cultured in RPMI‐1640 medium, supplemented with B27, heparin, N2 supplement, 20 ng/mL EGF and 20 ng/mL bFGF for 3 days to form spheres.

### 2.8 MTT assays

Cell viability was assayed using the CellTiter 96^®^ AQueous One Solution Cell Proliferation Assay (CellTiter96; Promega) according to the manufacturer's instructions. Briefly, the cells were seeded onto 96‐well plates and cultured for up to 7 days. At the end of each period, 10 μL MTT solution was added and the cells were incubated for an additional 4 hours, after which 150 μL dimethyl sulfoxide (DMSO) was added to each well and mixed thoroughly. The optical density of each well was measured with a spectrophotometer (UV5100, Shanghai).

### 2.9 5‐FU resistant cell generation

5‐FU resistant CRC cells were generated by continuous exposure to increasing concentrations of 5‐FU (from 5 to 30 μg/mL) with repeated subculture until fully resistant to 5‐FU. Cells were first cultured in growing medium with 5 μg/mL 5‐FU for 2 months, and the concentration of 5‐FU increased 5 μg/mL every 2 months.

### 2.10 Statistical analysis

Statistical analysis was performed using the GraphPad software (version 5.0). The differences between paired groups were analysed by Student's t‐test; differences between multiple groups were analysed by one‐way ANOVA. *P* values less than 0.05 were considered statistically significant. The data are expressed as mean ± standard error of the mean (SD).

## 3 RESULTS

### 3.1 Expression of the miR‐30‐5p family is decreased in CRC tissues and cell lines

**Figure 1:**
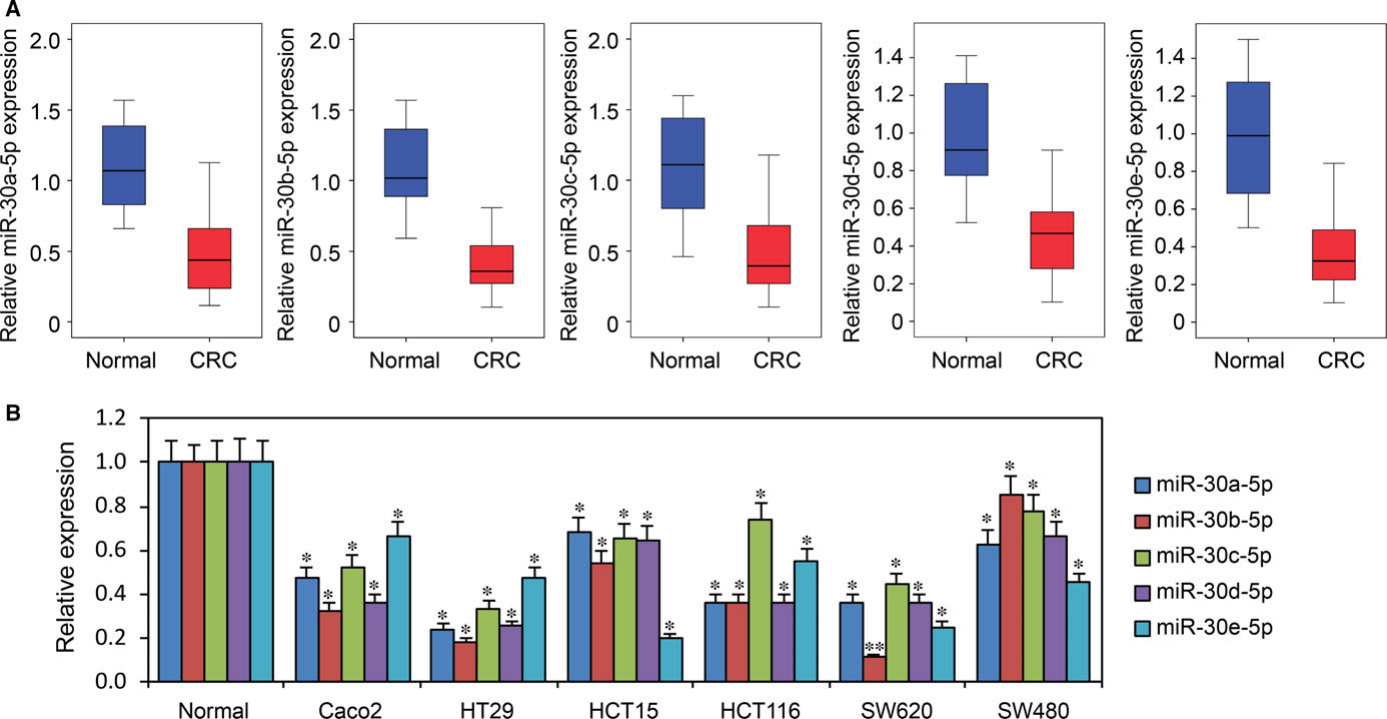
Expression of miR‐30‐5p family is decreased in CRC tissues and cell lines. A, MiR‐30‐5p family (miR‐30a‐5p, miR‐30b‐5p, miR‐30c‐5p, miR‐30d‐5p and miR‐30e‐5p) expression in 30 CRC tissues compared with adjacent normal tissues. *P *<* *0.001 compared with normal tissues. B, MiR‐30‐5p expression in normal CRC tissues and six CRC cell lines (Caco2, HT29, HCT15, HCT116, SW620 and SW480). *P <* *0.05 compared with normal CRC tissues

To investigate the miR‐30‐5p family (miR‐30a‐5p, miR‐30b‐5p, miR‐30c‐5p, miR‐30d‐5p and miR‐30e‐5p) function in CRC development, we first analysed miR‐30‐5p expression in paired primary CRC tissues and adjacent normal tissues from 30 CRC patients. Using RT‐PCR, we found that that miR‐30a‐5p, miR‐30b‐5p, miR‐30c‐5p, miR‐30d‐5p and miR‐30e‐5p were significantly decreased in primary tissues compared to normal tissues (Figure 1A). We then performed RT‐PCR for miR‐30‐5p in six CRC cell lines (Caco2, HT29, HCT15, HCT116, SW620 and SW480) and found that miR‐30‐5p expression was decreased in all cell lines compared with primary CRC tissues (Figure 1B).

### 3.2 MiR‐30‐5p directly targets USP22

**Figure 2:**
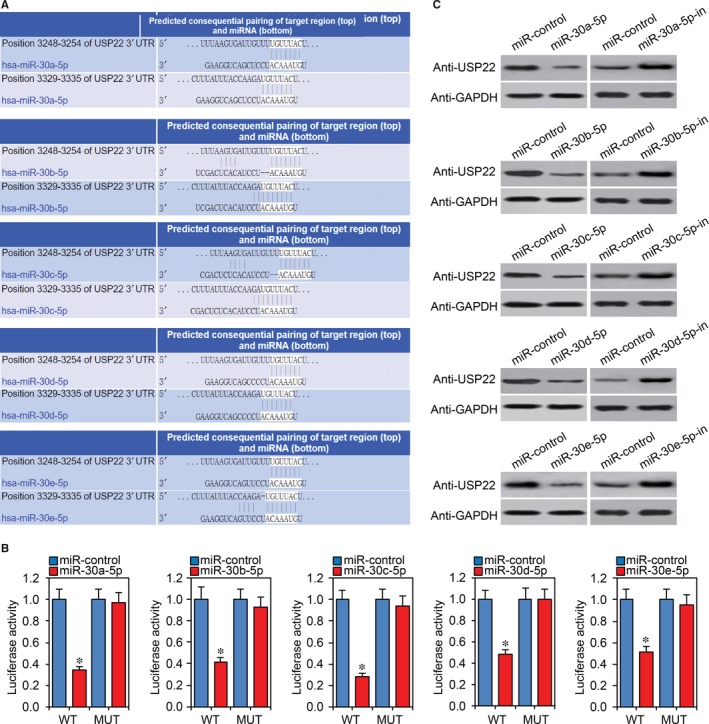
MiR‐30‐5p directly targets USP22. A Schematic representation of miR‐30‐5p binding sites in the USP22 3’UTR (SIP1 3’UTR WT). B, Luciferase activity in 293T cells co‐transfected with wild type (WT) or mutant (MUT) USP22 3’UTR luciferase reporter plasmid and miR‐30‐5p. *P <* *0.05 compared with miR‐control. C, USP22 protein levels assessed by western blotting in Caco2 cells transiently transfected with miR‐control, miR‐30‐5p or miR‐30‐5p inhibitor (miR‐30‐5p‐in)

To identify the target of miR‐30‐5p, the TargetScan target prediction algorithm was used to predict putative target genes with the miR‐30‐5p seed region sequence. The 3'UTR of USP22 was predicted to be a binding site of miR‐30‐5p (Figure 2A). In our previous report, we found that USP22 plays an important role in CRC development.^23^ We therefore investigated whether USP22 is the target of miR‐30‐5p. A luciferase reporter plasmid of wild type USP22 3'UTR containing the potential miR‐30‐5p binding sites or mutant was transfected into HEK293T cells along with miR‐30‐5p or control miRNA. MiR‐30‐5p significantly decreased the luciferase activity of wild type USP22 3'UTR and failed to affect mutant USP22 3'UTR (Figure 2B). We then transfected miR‐30‐5p, miR‐30‐5p inhibitor or control miRNA into Caco2 cells and found that miR‐30‐5p significantly reduced USP22 protein expression and miR‐30‐5p inhibitor promoted its expression (Figure 2C).

### 3.3 MiR‐30‐5p is decreased in CRC stem cells and overexpression of miR‐30‐5p reduces CRC cell stemness

**Figure 3:**
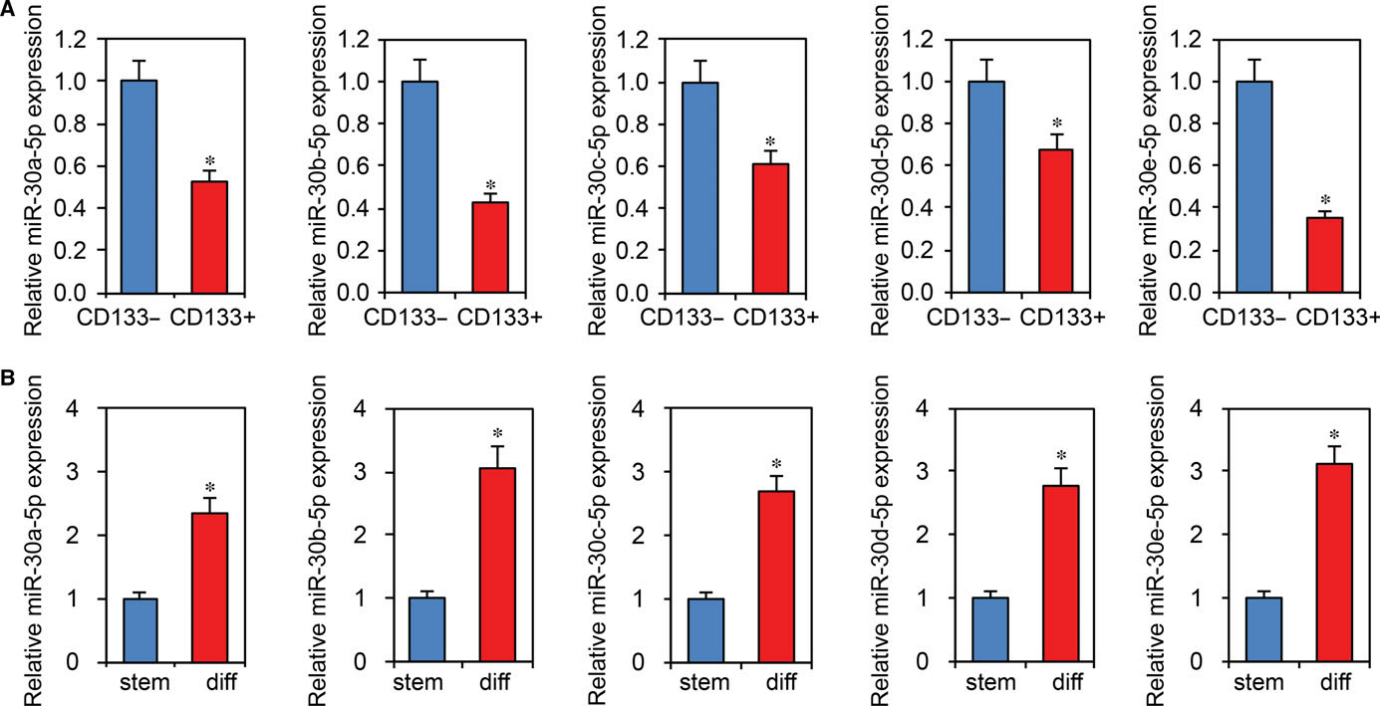
MiR‐30‐5p is decreased in CRC stem cells. A, RT‐PCR analysis of miR‐30‐5p in CD133‐ and CD133 + Caco2 stem cells. *P <* *0.05 compared with CD133‐ cells. B, HCT15 CD133 + stem cells were induced to differentiate. RT‐PCR analysis of miR‐30‐5p levels was assessed before and after cell differentiation. *P <* *0.05 compared with stem cells

**Figure 4:**
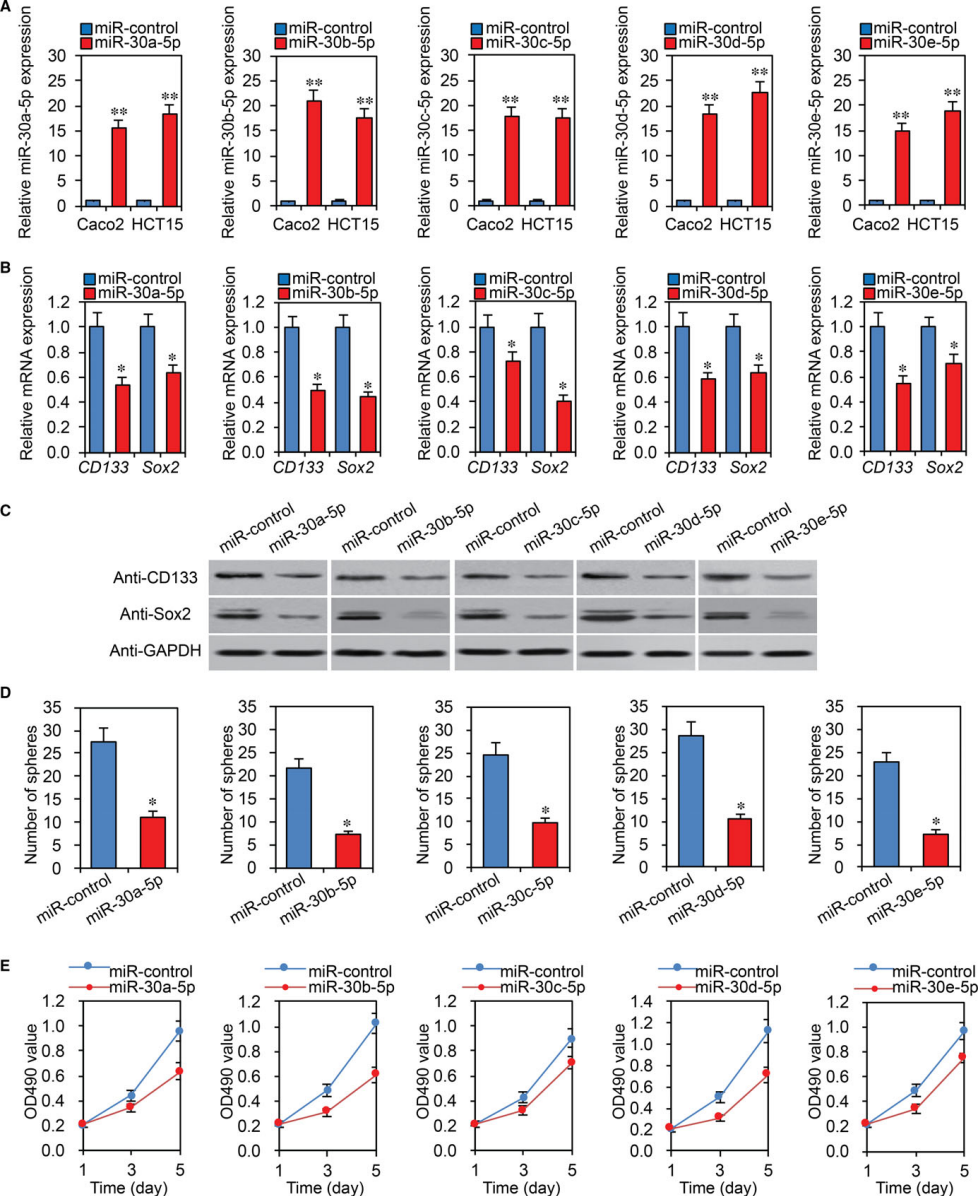
Overexpression of miR‐30‐5p reduces CRC cell stemness. A, MiR‐30‐5p levels assessed by RT‐PCR in Caco2 and HCT15 stem cells transiently transfected with miR‐control or miR‐30‐5p. **P < 0.01 compared with miR‐control. B, C, MiR‐30‐5p overexpression cells were subjected to RT‐PCR (B) and western blot (C) for CD133 and Sox2 mRNA and protein expression, respectively. *P <* *0.05 compared with miR‐control. D, MiR‐30‐5p overexpression Caco2 stem cells were subjected to sphere formation assays. The number of spheres was quantified. *P <* *0.05 compared with miR‐control cells. E, MTT analysis of miR‐30‐5p overexpression HCT15 stem cells

Previously, we demonstrated that USP22 maintains stemness in CRC stem cells.^23^ To evaluate the effects of miR‐30‐5p on CRC cell stemness, we isolated CD133 + Caco2 and HCT15 cells using microbeads.^23^ As shown in Figure 3A, miR‐30‐5p was significantly downregulated in CD133 + Caco2 stem cells. After inducing CD133 + HCT15 stem cells to differentiate, we observed that they also had decreased miR‐30‐5p expression (Figure 3B). To further study the role of miR‐30‐5p in CRC cell stemness, we transfected miR‐30‐5p into Caco2 and HCT15 stem cells. RT‐PCR confirmed the transfection's effectiveness (Figure 4A). Using RT‐PCR and western blot, we found that miR‐30‐5p transfection significantly decreased expression of stem cell markers CD133 and Sox2 in Caco2 and HCT15 stem cells (Figure 4B,C). We then performed sphere formation assays using miR‐30‐5p overexpression Caco2 cells. As shown in Figure 4D, miR‐30‐5p overexpression reduced the number of CD133 + Caco2 cell spheres. Furthermore, miR‐30‐5p overexpression significantly inhibited HCT15 stem cell proliferation, as determined by MTT assays (Figure 4E). In addition, we performed above experiments using miR‐30‐5p inhibitor. Opposite results were observed in CRC cells (Supplemental Figure S1).

### 3.4 Overexpression of miR‐30‐5p inhibits chemoresistance in CRC cells

**Figure 5:**
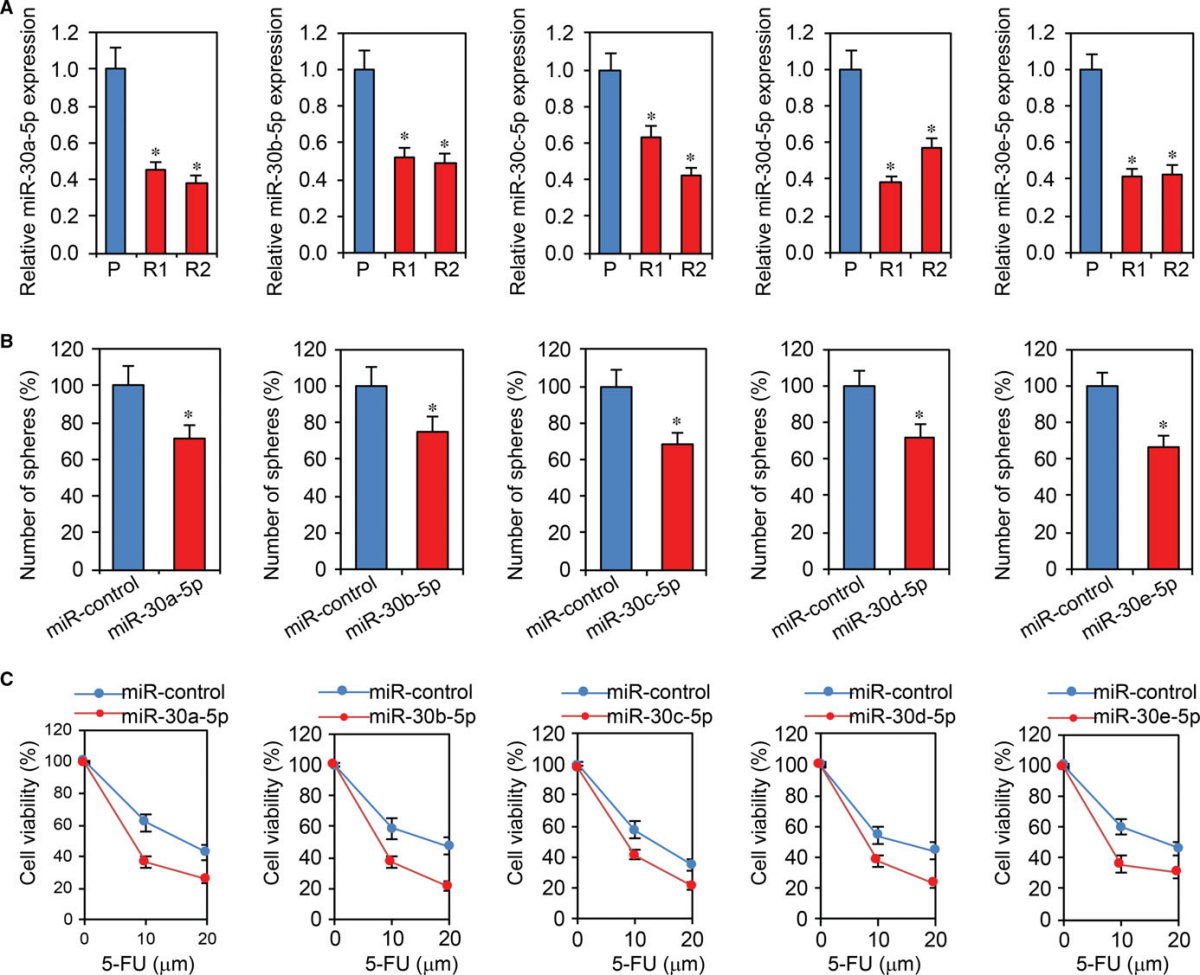
Overexpression of miR‐30‐5p inhibits chemoresistance in CRC cells. A, RT‐PCR analysis of miR‐30‐5p levels in 5‐FU resistant Caco2 cells. *P <* *0.05 compared with primary cells. B, Sphere formation assays in 5‐FU resistant Caco2 cells with miR‐30‐5p overexpression. *P <* *0.05 compared with miR‐control cells. C, Cell viability assays in 5‐FU resistant Caco2 cells with miR‐30‐5p overexpression

Our previous results demonstrated that USP22 is required for CRC cell chemoresistance.^23^ We therefore sought to determine whether miR‐30‐5p inhibits CRC cell chemoresistance by targeting USP22. We generated 5‐FU resistant Caco2 cells,^23^ and RT‐PCR showed that miR‐30‐5p expression was decreased in these cells (Figure 5A). We then increased USP22 expression in Caco2 and HCT15 cells with 5‐FU resistance. Sphere formation and cell viability assays revealed that overexpression of USP22 significantly reduced the rate of sphere formation and viability of CRC cells (Figure 5B,C). We further examined cell chemoresistance by miR‐30‐5p transfection and found inhibition of miR‐30‐5p promoted CRC chemoresistance (Supplemental Figure S2).

### 3.5 MiR‐30‐5p regulates Wnt/β‐catenin signaling pathway

**Figure 6:**
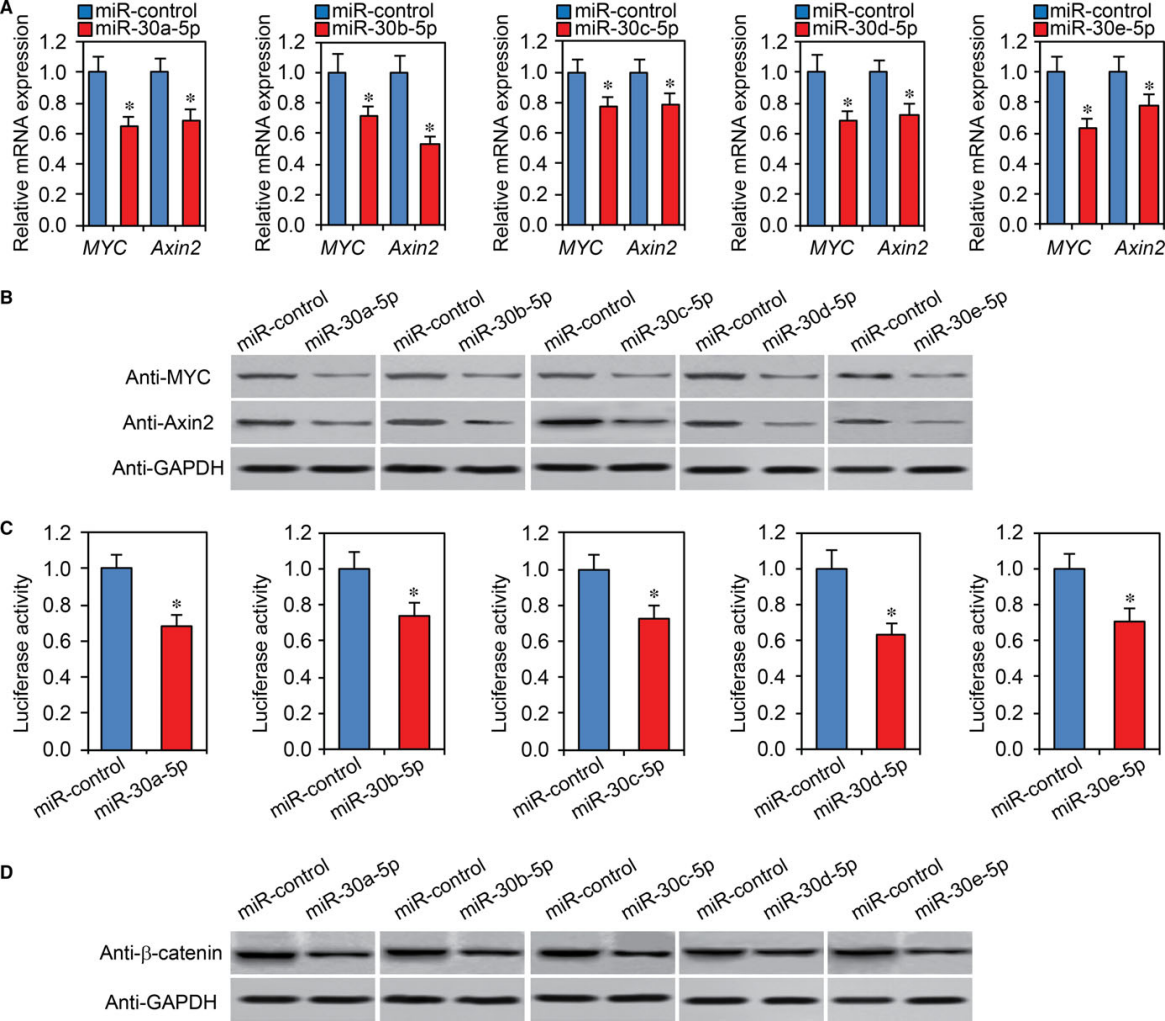
MiR‐30‐5p regulates the Wnt/β‐catenin signaling pathway. A, B, RT‐PCR (A) and western blot (B) analysis of Wnt/β‐catenin signaling target gene mRNA and protein levels in miR‐30‐5p overexpression CRC cells. *P <* *0.05 compared with miR‐control cells. C, Wnt luciferase analysis was performed in miR‐30‐5p overexpression Caco2 stem cells. *P <* *0.05 compared with control cells. D, Western blot analysis of β‐catenin levels in miR‐30‐5p overexpression HCT15 cells

Because USP22 regulates CRC stemness and chemoresistance via the Wnt/β‐catenin signaling pathway,^23^ we explored whether miR‐30‐5p affects CRC cell stemness and tumorigenesis through the same pathway. We performed RT‐PCR and western blot assays for Wnt/β‐catenin signaling target genes (Axin2 and MYC) in miR‐30‐5p overexpression Caco2 and HCT15 stem cells. As shown in Figure 6A,B, miR‐30‐5p significantly downregulated expression of these genes. Furthermore, Wnt luciferase activity assays in miR‐30‐5p overexpression Caco2 stem cells showed that miR‐30‐5p overexpression attenuated Wnt luciferase activity (Figure 6C). In HCT15 stem cells with miR‐30‐5p, a decrease of total β‐catenin protein levels was also observed (Figure 6D). Inhibitory results of miR‐30‐5p further confirmed regulation of Wnt/β‐catenin signaling pathway by miR‐30‐5p (Supplemental Figure S3).

### 3.6 MiR‐30‐5p regulates CRC cells through USP22

**Figure 7:**
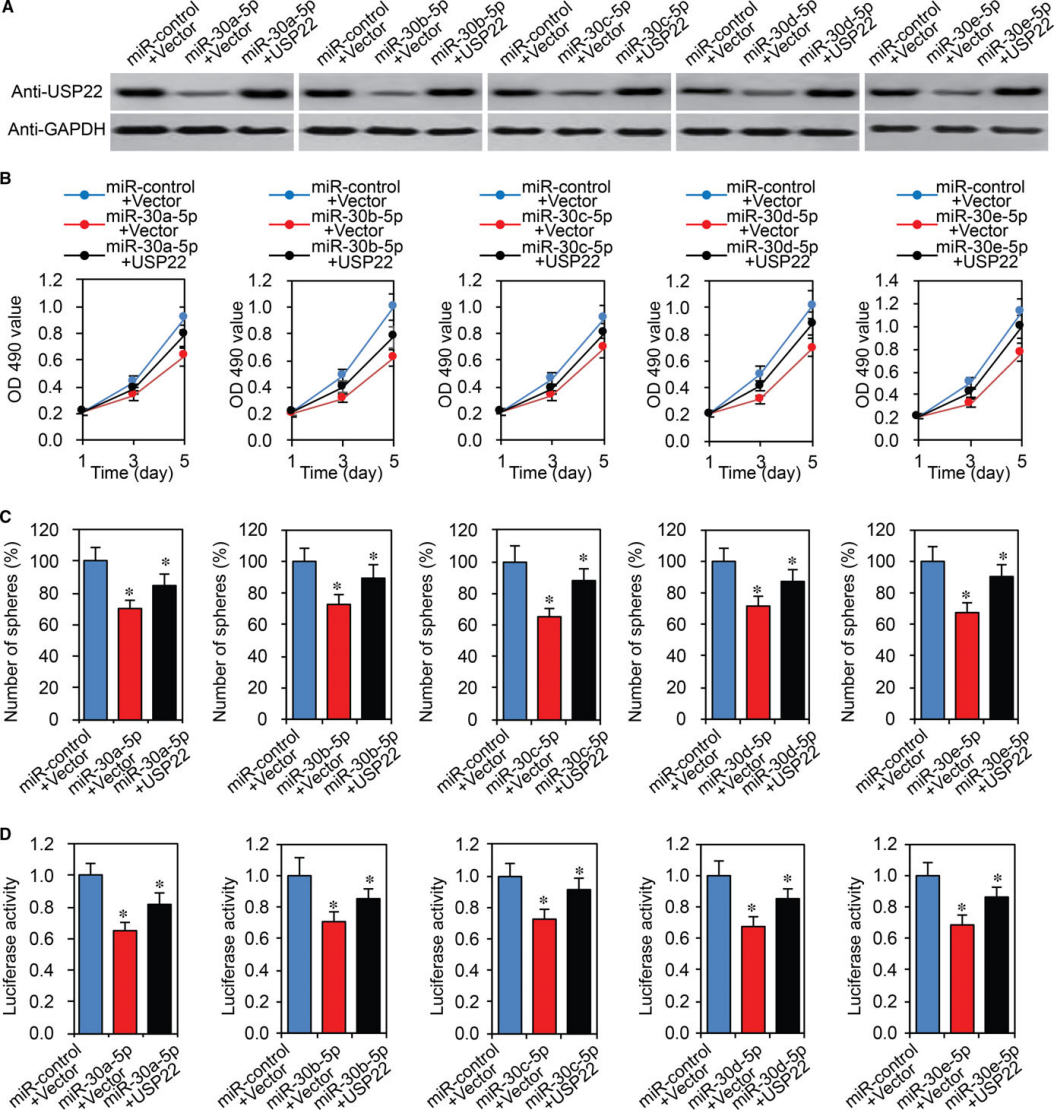
USP22 rescues the inhibitory effects of miR‐30‐5p on CRC cells. A, USP22 protein expression in Caco2 stem cells transfected with miR‐30‐5p or miR‐control and with or without the USP22 overexpression plasmid by western blot. B‐E, MTT, tumor sphere and Wnt luciferase activity assays in Caco2 stem cells transfected with miR‐30‐5p or miR‐control and with or without the USP22 overexpression plasmid. *P <* *0.05

To investigate whether miR‐30‐5p plays its function by targeting USP22 in the CRC cells, a rescue experiment was performed to analyse whether USP22 was involved in the miR‐30‐5p‐mediated malignant phenotypes of CRC cells. Caco2 stem cells were transfected miR‐30‐5p or miR‐control with USP22 overexpression plasmid. Western blot assay was used to confirm USP22 expression (Figure 7A). MTT assays showed that co‐transfection with the USP22 successfully rescued cell proliferation reduced by miR‐30‐5p (Figure 7B). We performed tumor sphere assays and found that the inhibited chemoresistance by miR‐30‐5p in CRC cells was partially abolished by USP22 overexpression (Figure 7C). Next, we performed TOPflash luciferase assays. As shown in Figure 7D, inhibited effect of luciferase by miR‐30‐5p was partially reversed by USP22.

## 4 DISCUSSION

In the present study, we have identified miR‐30‐5p as a crucial negative regulator of CRC stemness and chemoresistance induced by the USP22/Wnt/β‐catenin signaling axis. We began this study with the hypothesis that the mature miR‐30‐5p strand of precursor miR‐30 would serve an anti‐oncogenic role in CRC. By evaluating the expression levels of the miR‐30‐5p family in primary CRC tissues and cell lines, we substantiated this hypothesis, finding that miR‐30‐5p was decreased in both cases. The biological target prediction algorithm TargetScan then identified the 3'UTR of USP22 as a potential binding site of miR‐30‐5p. Given our prior study,^23^ in which we identified the USP22/Wnt/β‐catenin signaling axis as a promoter of CRC stemness and chemoresistance, we investigated the impact of miR‐30‐5p expression on levels of USP22, Wnt pathway target genes, and β‐catenin in CRC cells and 5‐FU resistant CRC cells. Not only did we find that miR‐30‐5p reduced expression levels through the USP22/Wnt/β‐catenin signaling axis, but we also determined that by doing so it decreases CRC phenotypic severity.

Stemness and chemoresistance are two major barriers to advancing CRC treatment regimens and represent the most pressing limitation for curing advanced disease.^33^ Beyond the individual harmful effects of stemness and chemoresistance, there is increasing evidence that they perpetuate each other. The theory that chemoresistance arises from cancer stem cells (CSCs) and then provides a more favorable milieu for CSCs to propagate is well supported for a variety of cancers, including CRC.^34^, ^35^, ^36^ Drug resistance can be acquired, inherent, or can result from a combination of both.^37^ Because CSCs are slow‐cycling, have superior DNA repair abilityand express ATP‐binding cassette (ABC) transporters that cause drug efflux, they give rise to inherent drug resistance.^38^, ^39^ Acquired resistance likely occurs when CSC subpopulations that survive a course of chemotherapy accumulate mutations that confer a chemoresistant phenotype.^40^ In both cases, increases in CSC populations and chemoresistance occur through a variety of deregulated signaling pathways, including Hedgehog/TGF‐β, EGFand Wnt/β‐catenin.^37^, ^39^, ^41^ In CRC specifically, the Wnt signaling pathway plays an outsized role in disease progression, with loss of Wnt pathway negative regulator adenomatous polyposis coli (APC) serving as a hallmark of human CRC, with more than 80% of patients having such mutations.^42^, ^43^ Therefore, any curative first‐line therapeutic for CRC will likely need to target the Wnt pathway.

To our knowledge, the present study is the first to investigate the role of miR‐30‐5p as a negative regulator of the Wnt/β‐catenin pathway in CRC. In our previous study,^23^ we determined that USP22 overexpression significantly enhances CRC stemness and chemoresistance by promoting Wnt/β‐catenin activity. At the molecular level, USP22 causes β‐catenin localization, which is ultimately necessary for the expression of Wnt target genes.^26^, ^44^, ^45^ In normal physiological states, Wnt signaling is activated at the base of intestinal crypts to maintain stem cell populations and intestinal epithelium homeostasis.^46^ When the pathway is aberrantly upregulated, however, both nonhypermutated microsatellite stable (MSS) and hypermutated microsatellite instability (MSI) CRCs can arise.^47^ Even though aberrant Wnt signaling is extremely prominent in CRC progression, given its importance in normal physiology, future studies that explore the use of exogenous miR‐30‐5p as a treatment should consider the appropriate amount to deliver without interfering with normal intestinal functioning. In addition to the heightened oncogenic gene expression caused directly by Wnt pathway overactivation, there are stepwise accumulations of gene mutations, such as *TGF‐*β*, p53*and *PI3K*, which cause cancer progression.^47^, ^48^ Targeting the USP22/Wnt/β‐catenin pathway should therefore be considered as one component of a more comprehensive treatment strategy.

## 5 CONCLUSIONS

MiRNAs are increasingly being appreciated for their roles in CRC as prognostic biomarkers and treatment possibilities. Given the significant challenges that aggressive phenotypes, including stemness and chemoresistance, pose in CRC, the use of exogenous miRNAs to target oncogenic signaling pathways in a multifactorial manner is a promising avenue for augmenting current treatment regimens. While future studies are necessary to determine miR‐30‐5p's range of effects in pathological and normal physiological states, the present study provides compelling evidence that miR‐30‐5p provides an effective means of targeting the oncogenic USP22/Wnt/β‐catenin signaling axis.

## CONFLICTS OF INTEREST

All authors declared no conflicts of interest in this work.

## AUTHOR CONTRIBUTIONS

SJ and JT designed the study. SJ, DM, MW, JL and YW performed the research. SJ, DM, MW and JT analysed the data. SJ, DM and JT wrote the manuscript. All authors read and approved the fnal manuscript. All authors read and approved the final manuscript.
