# Supplementary figures and images for: MiR‐30‐5p suppresses cell chemoresistance and stemness in colorectal cancer through USP22/Wnt/β‐catenin signaling axis
Source: J Cell Mol Med. 2018 Oct 19;23(1):630–40. doi: 10.1111/jcmm.13968 (PMC6307779; doi:10.1111/jcmm.13968)

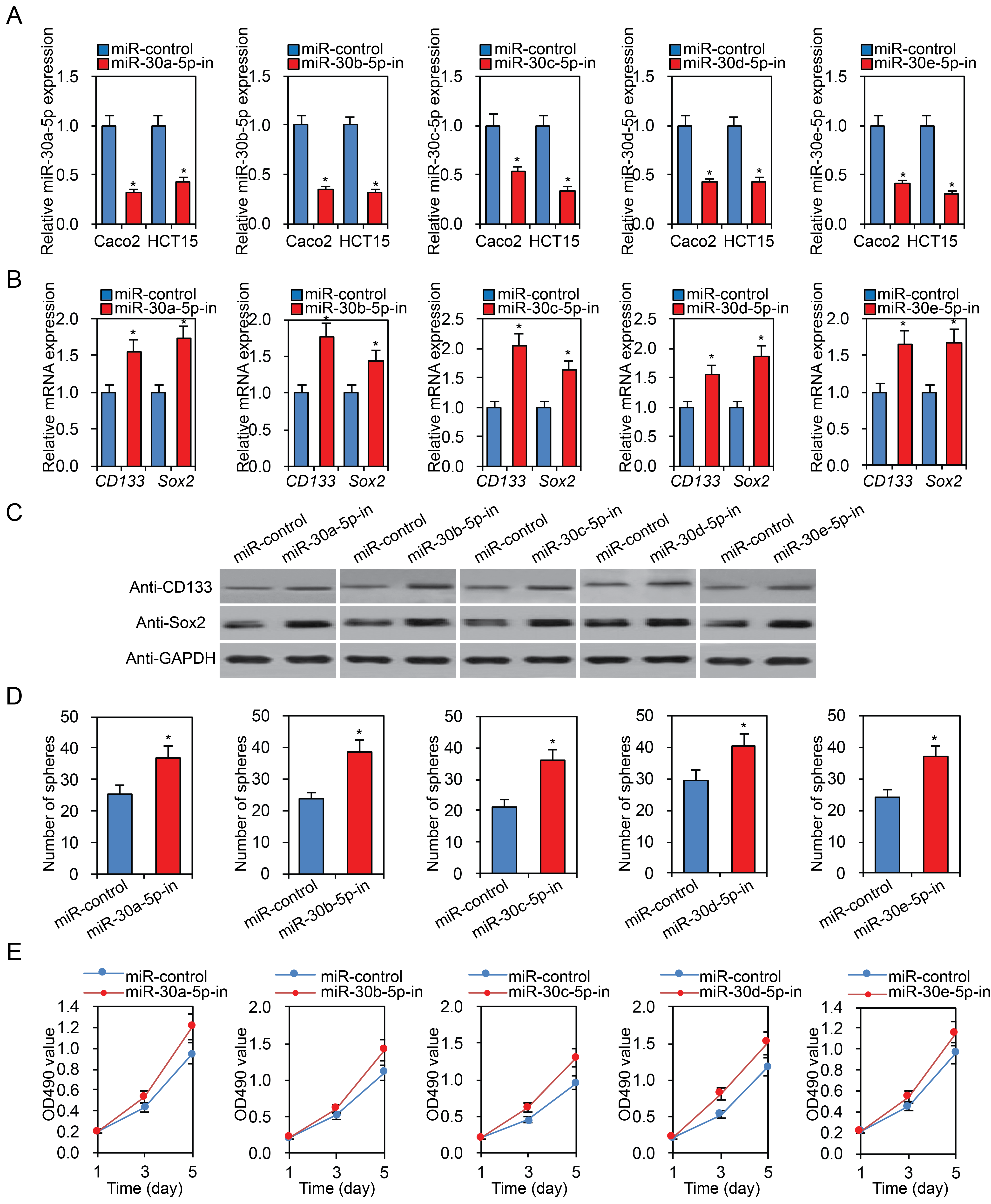

Supplement: Supplementary file 1 [file JCMM-23-630-s001.tif]

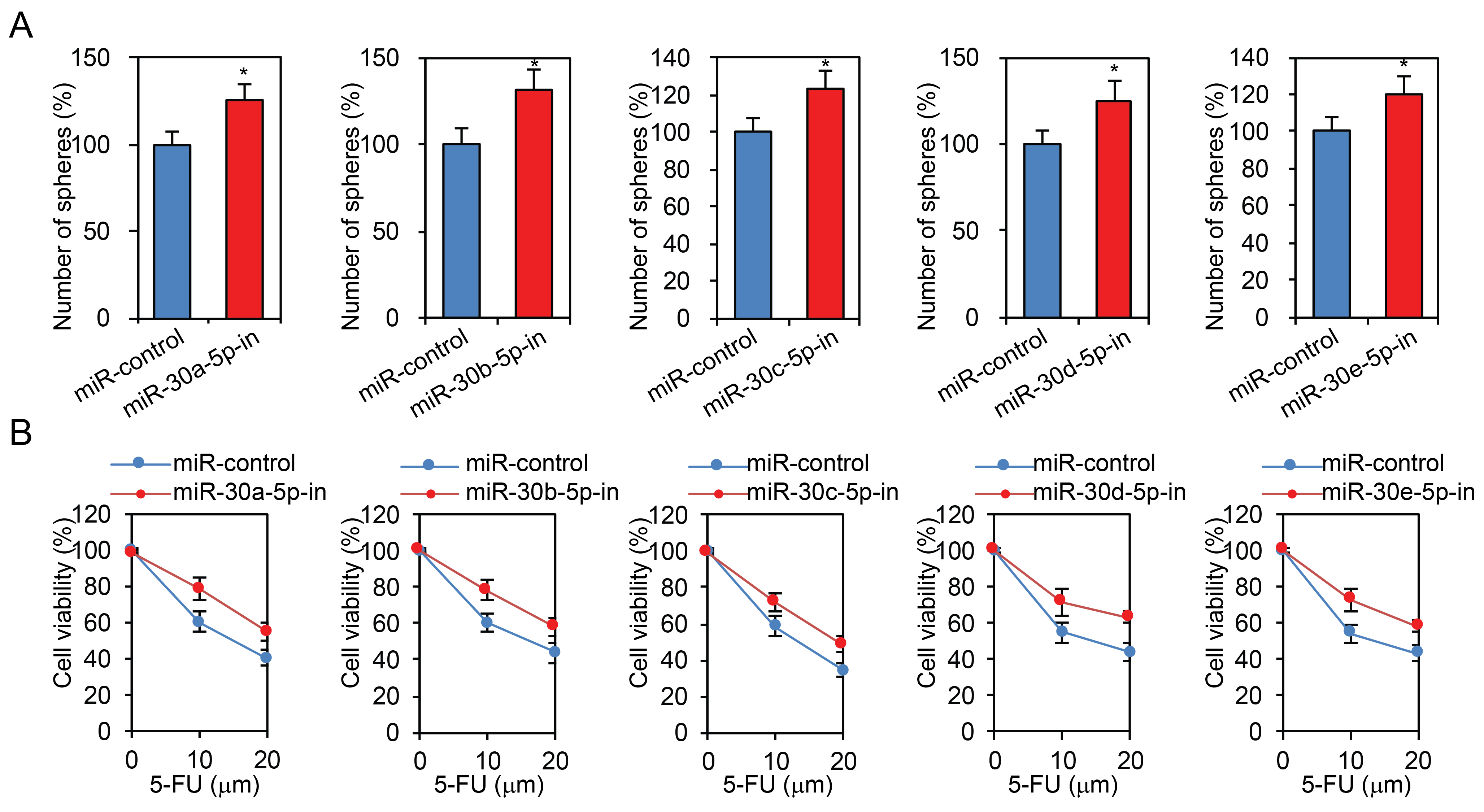

Supplement: Supplementary file 2 [file JCMM-23-630-s002.tif]

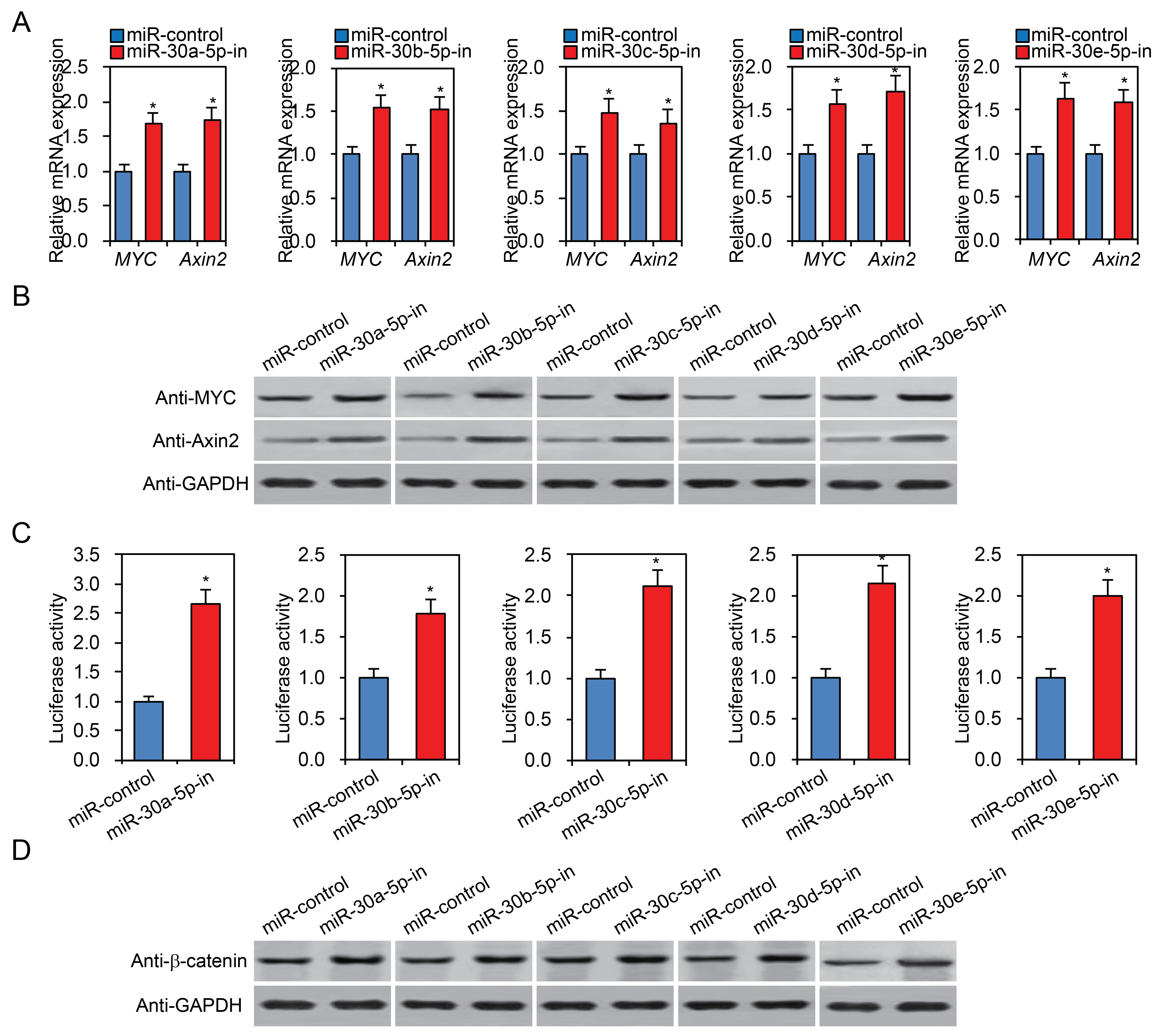

Supplement: Supplementary file 3 [file JCMM-23-630-s003.tif]
